# Better Together: Technology-Use Gaps and Loneliness in Older Adults

**DOI:** 10.3390/bs16091473

**Published:** 2026-08-24

**Authors:** Ortal Cohen Elimelech, Naor Demeter, Sara Rosenblum

**Affiliations:** 1 The Laboratory of Complex Human Activity and Participation, Department of Occupational Therapy, University of Haifa, Haifa 3103301, Israel; rosens@univ.haifa.ac.il; 2 Department of Occupational Therapy, Faculty of Social Welfare and Health Sciences, University of Haifa, Haifa 3103301, Israel; demeter@welfare.haifa.ac.il

**Keywords:** loneliness, older adults, technology-use gaps, structural equation modeling (SEM)

## Abstract

Loneliness is a growing public health concern among older adults, underscoring the need to better understand the complex interplay of factors associated with it. Although technology can enhance social connectedness, limited attention has been given to discrepancies between desired and actual technology use in daily life. This study examined loneliness, focusing on perceived technology-use gaps alongside health-related factors, including depressive symptoms, sleep quality, functional cognition, and sensorimotor abilities. Participants included 211 adults aged 65 to 87, who completed the de Jong-Gierveld Loneliness Scale, Geriatric Depression Scale, Pittsburgh Sleep Quality Index, Daily Living Questionnaire, selected items from the Washington Group Short Set on Functioning-Enhanced, and the Daily Technology-Use Gap questionnaire. Spearman correlations and structural equation modeling (SEM) were conducted. Loneliness was significantly associated with all variables (*r* = −0.64 to −0.32, *p* < 0.01–0.001). The SEM demonstrated good fit (comparative fit index = 0.986, root mean square error of approximation = 0.068). Depressive symptoms were significantly associated with loneliness, whereas technology-use gaps were identified as significant mediators in the proposed SEM, linking health-related factors and loneliness. These findings highlight a shift from focusing on technology access to understanding the quality and satisfaction of technology use in daily life.

## 1. Introduction

Loneliness is a subjective experience, often involving negative emotions. It arises from a discrepancy between an individual’s existing social relations and those they wish to have (Dykstra, 2009). Conceptualizations of loneliness commonly distinguish between two primary dimensions. Emotional loneliness refers to the absence of close, intimate, or emotionally supportive relationships. Social loneliness reflects a lack of broader social integration, such as meaningful group memberships or a cohesive social network (de Jong-Gierveld & van Tilburg, 2006). Although measuring the exact prevalence of loneliness among older adults is challenging, existing evidence has indicated that its prevalence is high enough to constitute a major public health concern (Schroyen et al., 2023; Surkalim et al., 2022).

In Israel, the prevalence of loneliness among adults aged 65 and older was approximately 27% in 2022, a figure higher than that observed among younger populations (Israel Central Bureau of Statistics, 2022). In North America, similar patterns have been reported, with approximately one-third of older adults experiencing loneliness, compared to substantially lower rates among younger populations (Salari et al., 2025). In the context of global population aging and increasing life expectancy (World Health Organization, 2022), these findings underscore the need to better understand the health consequences and underlying processes of loneliness.

From an evolutionary perspective, when loneliness persists rather than resolves, it may initiate changes across multiple domains essential for health and functioning in later life (Ong et al., 2016). Cacioppo’s evolutionary theory of loneliness provides a theoretical framework for understanding these processes (Cacioppo et al., 2014). According to this theory, human beings possess a fundamental drive for social connection, and loneliness functions as an adaptive signal alerting individuals to the need to improve social connections (Cacioppo & Patrick, 2008). However, when this signal becomes chronic, it may adversely affect immune and cardiovascular functioning, potentially accelerating biological aging processes (Ong et al., 2016). As such, emotional and social loneliness reflect unmet relational needs at different levels of social connection. Building on this framework, the present study examines key relationships among loneliness and depression, sleep quality, cognitive functioning, sensorimotor abilities, and technology use.

Prior longitudinal studies have identified health mechanisms related to loneliness among older adults, including its association with depression (Dahlberg et al., 2022), sleep disturbances, and impaired cognitive abilities (Cachón-Alonso et al., 2023; Kwak et al., 2023; Qi et al., 2023). Given their significance for health and implications for daily functioning (Rosenblum & Elimelech, 2021), these factors have received substantial research attention. For instance, loneliness and depression are often interrelated (Alpass & Neville, 2003; Dahlberg et al., 2022). Although loneliness is often linked to the onset of depressive symptoms, older adults often perceive it as a key contributing factor to their mental decline (Barg et al., 2006). In addition, a systematic review has highlighted the bidirectional relationship between loneliness and sleep, suggesting that loneliness intensifies feelings of vulnerability and stress, which in turn disrupt sleep quality (Deng et al., 2023). Furthermore, poor sleep quality has been linked to both cognitive and emotional difficulties, including reduced working memory performance and increased depressive symptoms, highlighting its potential relevance to the experience of loneliness (Federico et al., 2022).

In parallel, research examining cognitive functioning, including recall, processing speed, and memory, consistently demonstrates an association between loneliness and poorer cognitive performance in later life, despite variability in measurement approaches across studies (Boss et al., 2015). More specifically, self-reported difficulties in everyday cognitive functioning have been found to be related to experiences of loneliness (Juras et al., 2025). In everyday life, there is a continuous process of cognitive problem-solving to meet functional demands essential to maintaining independence (Giles et al., 2020). In social contexts, these abilities are vital for maintaining a conversation in noisy environments and for initiating and organizing social gatherings (Donovan et al., 2008). To better capture these everyday cognitive demands, the present study focuses on functional cognition, defined as the application of cognitive and performance skills (e.g., motor skills that facilitate action) to execute daily activities (Giles et al., 2020).

Coupled with functional cognition, sensorimotor abilities constitute another essential domain supporting effective communication and social participation (Buchman et al., 2009; Harithasan et al., 2020). These abilities—including vision, hearing, and fine motor skills—frequently deteriorate with age (He et al., 2017) and have been closely linked to increased feelings of loneliness (Wang et al., 2022). Examining both domains together may therefore deepen the understanding of the mechanisms underlying loneliness in later life (World Health Organization, 2022), with important implications for identifying intervention targets among older adults.

Building on this understanding, technology is one such avenue, offering possibilities to strengthen social connections among older adults. Technology use—encompassing common devices such as smartphones, computers, and tablets—has fundamentally transformed how people communicate, connect, and maintain relationships (World Health Organization, 2022; Yeo et al., 2024). Extensive research has demonstrated that engaging with such devices provides substantial health benefits, particularly in addressing social isolation in later life (Bertocchi et al., 2022; Kim et al., 2020; Umoh et al., 2023).

Beyond its influence on loneliness, technology use has also been examined in relation to broader outcomes, including depression, functional cognition, and sleep quality (Elliot et al., 2014; Iancu & Iancu, 2020; Shin et al., 2017). Importantly, the ability to benefit from technology depends in part on users’ sensorimotor abilities, which directly shape their capacity to interact with it (Elboim-Gabyzon et al., 2021). Taken together, these findings suggest that technology use may serve as a potential mediating mechanism—being associated with differences in the relationship between health-related factors and loneliness in older adults.

However, a closer examination of technology, loneliness, and its health-related relationships reveals a multifaceted complexity. Research has indicated that information and communication technology (ICT)—measured by email frequency and internet use for shopping, banking, and health purposes—was not directly related to depressive symptoms. Rather, ICT use mediates the relationship between limitations in activities of daily living (ADLs) and depressive symptoms (Elliot et al., 2014). Likewise, another study found that using technology for social interactions, such as email, social networking sites, and video calls, was correlated with better self-rated health. Interestingly, this relationship was fully mediated by reduced loneliness (Chopik, 2016). In line with this, a subsequent study found that the relationship between social technology use (e.g., communicating via Skype with family and friends) and loneliness was influenced by social engagement and frequency of social contact (Byrne et al., 2021).

Despite these promising associations, evidence regarding the effectiveness of technology-based approaches remains mixed. A recent meta-analysis found no conclusive evidence that digital interventions effectively reduce loneliness among older adults (Shah et al., 2021). Digital interventions are broadly defined as applications of technology, equipment, and software that process information through numeric codes, encompassing a diverse array of tools, including web-based social networking platforms, sensors, and robotics (Barbosa Neves et al., 2019; Shah et al., 2021). These findings suggest that the association between technology use and loneliness may be shaped by health-related factors rather than by technology use alone. Accordingly, there is a need to move beyond examining technology use in isolation and instead investigate the dynamic interrelations among these factors and the complex role technology may play within them.

**Figure 1:**
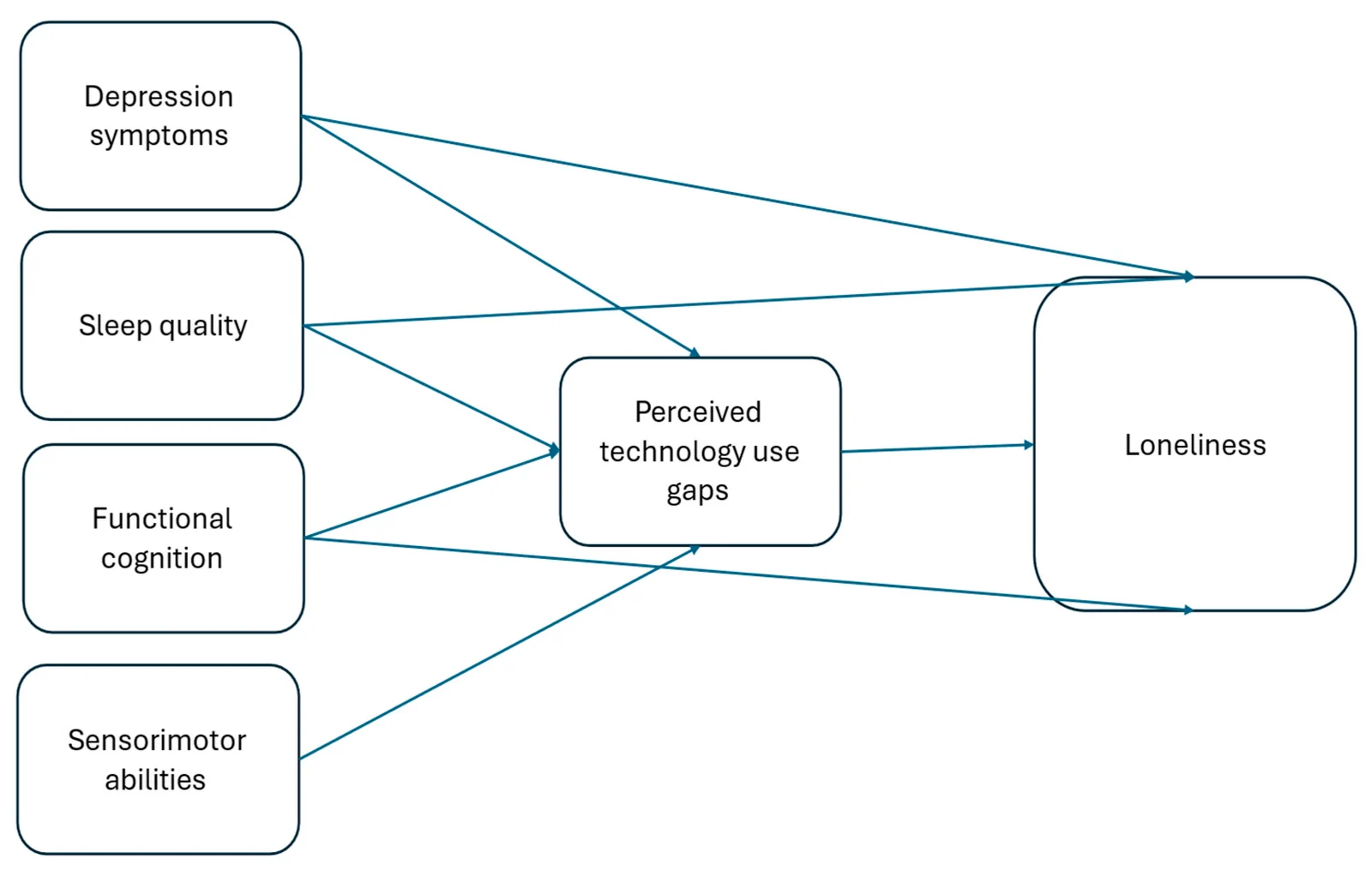
The proposed model illustrates the potential role of technology in mediating the relationship between health-related factors and loneliness.

To our knowledge, no study has examined technology use from the perspective of unmet needs in daily activities. Consequently, the gap between the activities an individual desires to accomplish with technology and their actual performance is often overlooked. This study introduces the concept of technology-use gaps as a distinct construct that reflects discrepancies between desired and actual technology use in daily life. Given the established relationship between engaging in meaningful activities and health (Ryu & Heo, 2018; Vagetti et al., 2014), this gap may represent an important area of inquiry. Therefore, this study aims to examine perceived loneliness, with an emphasis on understanding technology use characteristics within the complex interplay of health-related factors, including depression, sleep quality, functional cognition, and sensorimotor abilities. Figure 1 presents the proposed model, illustrating these potential relationships based on the theoretical framework and the previous literature described previously.

## 2. Materials and Methods

### 2.1. Procedure and Participants

This study received ethical approval from the University of Haifa Faculty Ethics Committee (Approval Number #285/23). Data collection was conducted during March 2024 using the Israeli survey company Panel4All. This company recruits participants for web-based research through digital advertisements, typically offering financial incentives. Approximately 400,000 members are recruited through digital advertisements on major platforms such as Google and Facebook, reaching a broad cross-section of the adult population in Israel. Panel4All recruited this study’s panelists via an invitation distributed to its registered members.

A total of 247 panelists were initially enrolled in this study. Of this group, 34 participants (14%) did not complete the questionnaire, and two participants (1%) were excluded for failing to meet the minimum age requirement. Consequently, the final analytic sample that met the inclusion criteria (aged 65 or older) consisted of 211 individuals aged 65 to 87. The proposed structural equation modeling (SEM) included 22 freely estimated parameters, yielding a participant-to-parameter ratio of approximately 9.6:1. Given that a ratio of approximately 10 participants per estimated parameter has been suggested as a general guideline for the SEM analyses (Schreiber et al., 2006), the sample size was considered adequate for the proposed model.

After receiving information regarding the study’s aims, procedures, and ethical considerations, participants provided informed consent and completed the study questionnaire via the Qualtrics platform (Qualtrics, Provo, UT, USA). The survey was conducted in Hebrew and took approximately 25 min to complete.

### 2.2. Instruments

- Demographic and health background questionnaire

This questionnaire included questions regarding age, gender, education, years since retirement, and chronic conditions.

- Loneliness

The De Jong-Gierveld Loneliness Scale (de Jong-Gierveld & Kamphuls, 1985) is a well-established self-report questionnaire that measures emotional and social loneliness. It consists of 11 items, with scales ranging from 1 (*yes*) to 5 (*no*). Five of the items evaluate feelings of belonging, and the other six measure feelings of social loss. The sum of the items was used to compute the loneliness score (de Jong-Gierveld & Kamphuls, 1985; de Jong Gierveld et al., 2009). This questionnaire has been shown to be reliable and valid in previous studies, including the Hebrew version (Iecovich, 2013).

- Depression

The Geriatric Depression Scale (GDS; (Sheikh & Yesavage, 1986) includes 15 self-report questions designed to measure depression among older adults. In general, a score greater than 5 indicates depressive symptoms, and a score between 0 and 5 indicates normal (Claims et al., 1994; Sheikh & Yesavage, 1986).

- Sleep quality

The Pittsburgh Sleep Quality Index (PSQI; (Buysse et al., 1989) is a self-report questionnaire for assessing sleep quality and disturbances that may have affected participants’ sleep quality in the past month. It consists of 18 items divided into seven components (e.g., sleep quality, sleep latency, sleep duration, habitual sleep efficiency). A summary score is calculated for each component; the seven component scores sum to the global PSQI score (range: 0–21). Higher scores indicate lower sleep quality (Buysse et al., 1989). The PSQI’s validity and reliability have been established (Wu et al., 2012).

- Functional cognition

The Daily Living Questionnaire (DLQ; (Rosenblum et al., 2017) is a 51-item self-report instrument that measures functional cognition and addresses cognitive difficulties related to daily functioning. The questionnaire is divided into two main parts: Part A (28 items) assesses activities and participation across four factors (household tasks, activities involving language/comprehension, community/participation, and complex tasks), and Part B (23 items) examines cognitive symptoms or impairments across three factors (executive function, memory, and executive function monitoring). Respondents rate their mental difficulty using a 4-point Likert scale from 1 (*no mental difficulty*) to 4 (*unable to complete*; (Rosenblum et al., 2017). The DLQ’s content and face validity have been established, as well as its internal consistency, and it can distinguish between different age groups and clinical diagnoses (Rosenblum & Elimelech, 2021).

- Sensorimotor abilities

Sensorimotor abilities were assessed using four questions adapted from the Washington Group Short Set on Functioning-Enhanced (WG-SS Enhanced; Washington Group, 2022). In the WG-SS Enhanced, eight essential domains of functioning are examined. The selected questions address vision, hearing, and upper body functions. Participants were asked to rate their level of difficulty for each item—vision, hearing, functional upper limb use, and use of hands and fingers—using a four-point ordinal scale: *no difficulty*, *some difficulty*, *a lot of difficulty*, and *cannot do at all*. The items were translated into Hebrew with permission from the authors.

The internal consistency for this section was α = 0.63, which may be partly attributable to the small number of items. To further examine the scale, we analyzed the item-total correlations, which were all significant (*p* < 0.001) and exceeded *r* > 0.62, indicating meaningful relationships between each item and the overall score (Tavakol & Dennick, 2011). In addition, the Spearman-Brown prophecy formula was applied to estimate the potential increase in reliability that would result from increasing the number of items (Warrens, 2015).

$$R_{spearman-Brown}=\;\frac{nr}{1+\left(n-1\right)r}$$

According to this formula (de Vet et al., 2017), doubling the number of items (*n* = 2) would be expected to increase reliability to α = 0.77, whereas tripling the number of items (*n* = 3) would be expected to increase reliability to α = 0.83. These findings suggest that the relatively modest internal consistency observed in the present study may be partly attributable to the small number of items included in this measure.

- Technology use characteristics

The Daily Technology-Use Gap (DTUG) is a self-reported questionnaire designed to analyze older adults’ technology use in relation to their daily activities. The questionnaire was developed specifically for this study, and its content validity was established by consensus among professionals experienced in working with older adults (*n* = 21) (Berkowsky et al., 2017).

The DTUG comprises three sections: technology use patterns, social support, and perceived technology gaps.

1. Technology use patterns

Given the wide variety of devices available, an emphasis was placed on three commonly used digital devices: smartphones, computers, and tablets. First, participants were asked to indicate which of these devices they owned (multiple responses were allowed) and how frequently they used them during the past month on a five-point Likert scale ranging from 1 (*not at all*) to 5 (*several times per day*). Each of the following items refers specifically to the device that the participant primarily utilizes. It covers the following domains:

(a) Perceived proficiency—Participants were asked to rate their self-perceived proficiency for the device on a scale from 1 (*not good*) to 5 (*excellent*).

(b) Adaptability—Participants were asked if they were interested in receiving guidance or instruction to improve their use of the device, using a 5-point scale from 1 (*not at all*) to 5 (*very much*).

2. Social support

Participants were asked to rate the extent to which they had access to individuals who could provide support with using the device, on a 5-point scale from 1 (*not at all*) to 5 (*very much*). Participants who selected *1* in this domain were instructed to skip the following questions. Those who indicated they were able to access social support were asked three additional questions regarding: (a) the level of assistance they received (e.g., explanations, demonstrations, advice) according to their needs based on a scale of 1 (*not at all*) to 5 (*very much*); (b) the person who usually assists them with options for their spouse, child, grandchild, friend, or other; and (c) their level of comfort in requesting such assistance, rated from 1 (*not at all*) to 5 (*very much*).

3. Perceived technology gap

This section explores participants’ use of digital devices (smartphones, computers, and/or tablets) across 19 daily activities (e.g., meal planning and preparation, managing health-related tasks, and physical activities). The resulting gap score is intended to capture the cumulative extent of unmet technology-related needs across everyday activities rather than difficulties within any specific activity domain. For each activity, participants were first asked whether they currently use any of the listed devices to perform the activity (*yes*/*no*/*not relevant*).

Participants who answered *yes* were asked to rate their satisfaction with their current use of the device for that specific activity. Satisfaction was rated on a four-point Likert scale ranging from 1 (*not satisfied at all*) to 4 (*very satisfied*). Participants who reported low or partial satisfaction (responses 1 or 2) were asked what they needed in order to utilize technology for the purpose of conducting the activity. For this question, the predefined list of potential needs included: knowledge of available applications relevant to the activity; knowledge or skills for utilizing the device for this purpose; technical improvements to make the device suitable for their needs; human support; or other (free-text response).

Participants who answered *no* (i.e., not using any of the devices for that activity) were asked whether they would be interested in using such technology for that purpose in the future (*yes*/*no*). If they answered *yes*, they were similarly asked to specify what would be required to enable such use.

This section enables the identification of two distinct types of technology-use gaps: (a) not currently using a device for an activity but expressing interest in doing so and (b) currently using the technology but reporting low or partial satisfaction with its use. Each activity identified by the participant as having a gap was coded as 1, and activities without reported gaps were coded as 0. A total gap score was then calculated by summing the number of gaps across 19 daily activities. Higher scores indicate a greater number of unmet technology-related needs across daily activities.

Internal consistency was examined for the overall DTUG gap score and for the two gap types separately. The overall DTUG gap score yielded an internal consistency estimate of α = 0.68. The internal consistency of both gap types was low (α = 0.55 for the non-use gap score; α = 0.66 for the dissatisfaction gap score). Given the heterogeneous nature of the activities included in the DTUG and the fact that the measure was designed to capture technology-use gaps across diverse domains of everyday life, these reliability estimates should be considered preliminary.

### 2.3. Data Analysis

Data were analyzed using IBM SPSS Statistics (IBM Corp., Armonk, NY, USA; version 30). Demographic characteristics (e.g., age, gender, years of education) were summarized using descriptive statistics. To examine the relationships between depression, sleep quality, functional cognition, sensorimotor abilities, and technology use (technology support and perceived technology gaps), a correlational analysis was conducted. Based on the distribution, Spearman’s rank-order correlation—a non-parametric test—was selected to examine these associations. Statistical significance was set at *p* < 0.05.

To test the proposed loneliness model, an SEM analysis was conducted using the IBM SPSS AMOS (IBM Corp, Version 25) program. Missing values were handled using the SPSS Series Mean procedure prior to the SEM estimation. The final SEM was estimated using the transformed variables, yielding complete data for the analytic sample (*N* = 211). The goodness of fit for the model was assessed using three primary criteria: the chi-square statistic (χ^2^), the comparative fit index, and Steiger’s root mean square error of approximation (Weston & Gore, 2006). A maximum likelihood estimation was used. Because the technology-use gap score represented a positively skewed count variable, with 41.2% of participants reporting no technology-use gaps, the maximum likelihood estimation was supplemented with 5000 bootstrap resamples and bias-corrected 95% confidence intervals (Lai, 2018). In addition, alternative model specifications were explored during model development, including models that incorporated both DLQ Parts A (activities and participation) and B (cognitive symptoms and impairments).

## 3. Results

### 3.1. Demographic Characteristics and Medical Status

Among the participants (*N* = 211), 49% were female (*n* = 104). The majority were married (74%), identified as secular (69%), and had completed more than 12 years of formal education (73%). Table 1 provides a detailed description of the sociodemographic characteristics of the sample.

Regarding medical status, approximately half of the participants (47%) reported having a cardiovascular disease. Additional diagnoses included diabetes (15%), thyroid deficiency (7%), cancer (6%), and neurological disorders (5%). The remaining participants (7%) reported musculoskeletal disorders, autoimmune diseases, chronic obstructive pulmonary disease, or fibromyalgia.

### 3.2. Loneliness, Depression, Sleep Quality, Functional Cognition, and Sensorimotor Abilities

For the De Jong-Gierveld Loneliness Scale, 43% of participants reported not experiencing loneliness, 39% reported being moderately lonely, 17% reported being lonely, and 1% reported being extremely lonely. According to the GDS, most participants (80%) scored between 0 and 4, which means depressive symptoms were typically not of concern. The GDS mean score was 2.47 (*SD* = 3.37).

The mean sleep quality, as measured by the PSQI, was 4.77 (*SD* = 2.81), which indicates that participants experienced minor sleep disturbances.

For functional cognition, as measured by the DLQ, the mean total score across all parts ranged from 1.15 to 1.30 (*SD* = 0.30–0.40), indicating that participants reported slight cognitive difficulties with activities and participation. Approximately 35% to 40% of participants reported cognitive difficulty on the following items: managing complex tasks requiring a large amount of information to be handled simultaneously (item 32; 40%), removing irrelevant background noise or thoughts while performing a task (item 33; 37%), and managing multi-step tasks (item 45; 35%). These three items, which comprise Part B of the DLQ, had the highest reported difficulty rates of all the items. The reliability of these items was reasonable (α = 0.72), with a mean score of 1.42 (*SD* = 0.49). It was found that the mean scores of these three items were significantly correlated with depression (*r* = 0.36, *p* = 0.001) and sleep quality (*r* = 0.32, *p =* 0.001).

Regarding the sensorimotor abilities, participants reported high levels of intact functioning. Across the four items reflecting these abilities, the mean score ranged from 1.23 to 1.28 (*SD* = 0.25–0.54).

The results for loneliness, depressive symptoms, sleep quality, cognitive function, and sensorimotor abilities are summarized in Table 2.

### 3.3. Technology Use Characteristics (DTUG Questionnaire)

1. Technology-use patterns

Participants were asked whether they owned each of the following devices: a smartphone, a computer, and a tablet. Most reported owning a smartphone (98%) and a computer (83%), whereas fewer owned a tablet (*n* = 84, 40%). Participants were then asked to identify the device they use most frequently. The majority reported using a smartphone most frequently (60%), followed by a computer (20%), and a tablet (5%); however, 15% of the sample did not respond to this question. In general, participants reported using their primary device several times per day (*M* = 4.95, *SD* = 0.23), reflecting high daily use. An overview of device ownership and usage patterns is provided in Table 3.

2. Social support

Regarding social support for technology use, participants reported relatively high availability of support (*M* = 3.49, *SD* = 1.26) and that support generally met their needs (*M* = 3.61, *SD* = 1.14). The main sources of support were sons and daughters (53%). Table 3 describes the characteristics of participants’ technology patterns and the social support they receive.

3. Perceived technology gap

**Figure 2:**
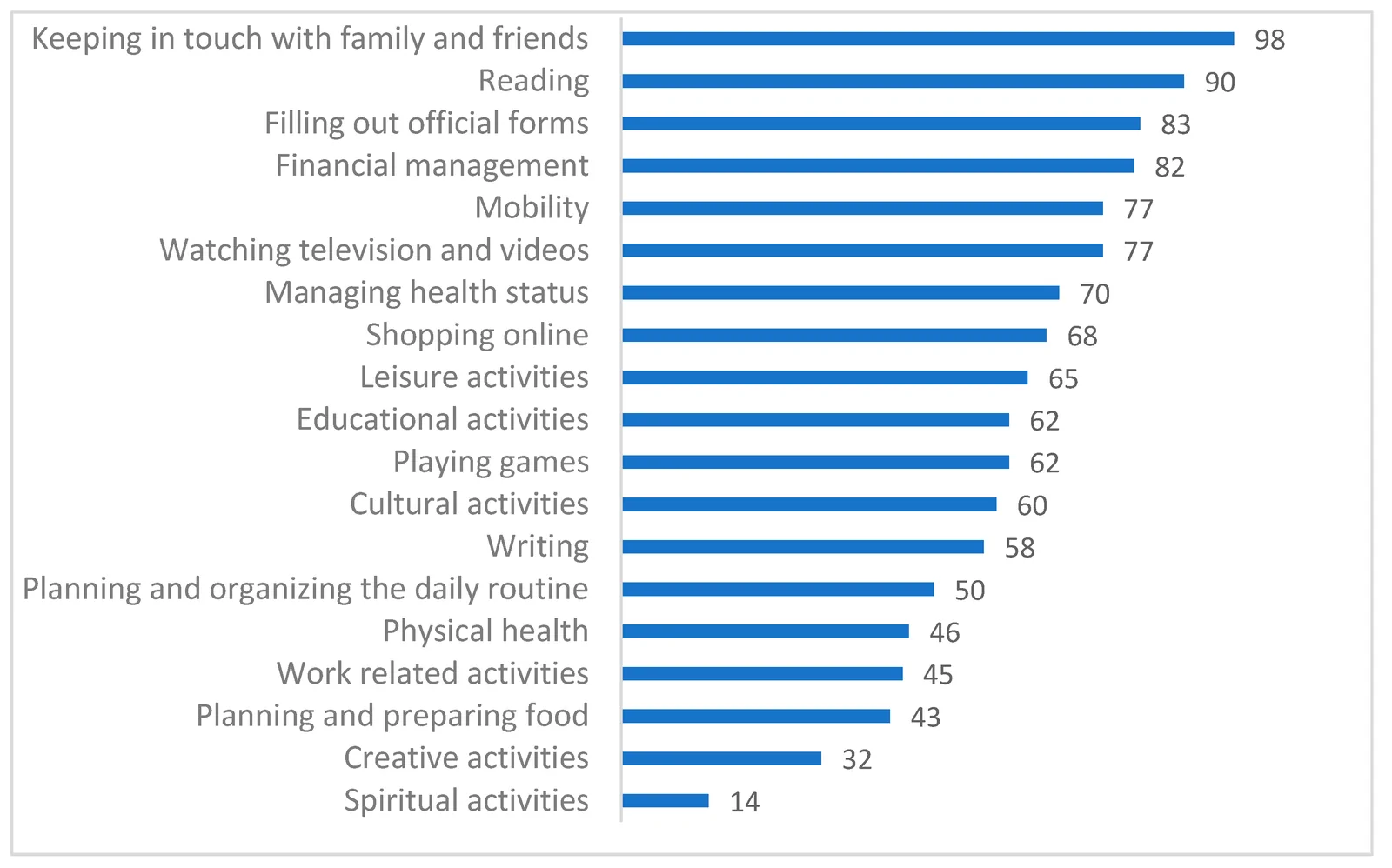
Use of primary technological devices across daily activities (% of participants).

Figure 2 presents the percentages of participants who reported engaging in various activities using their primary device.

**Figure 3:**
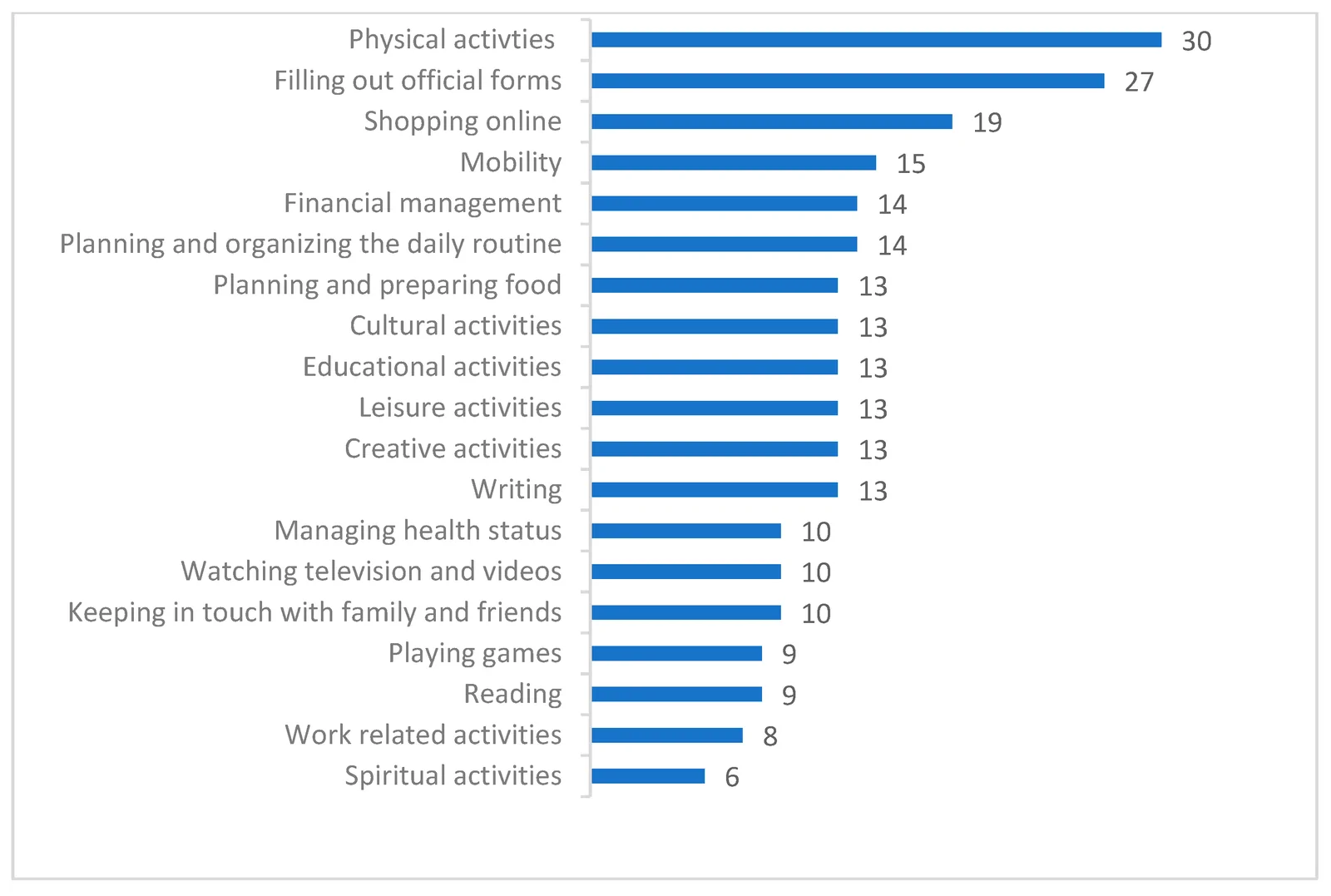
Percentages of technology-use gaps across different activities among participants who reported at least one gap (*N* = 124).

As shown, the majority indicated using technology to keep in touch with family and friends (98%), to read (90%), to fill out official forms (83%), and for financial management (82%). Two types of technology-use gaps were identified: (a) not engaging in a certain activity despite being interested in doing so and (b) engaging in the activity but not being satisfied with the experience. The mean total gap score was 1.29 (*SD* = 1.67), ranging from 0 to 9. Figure 3 presents the distribution of these gaps across different activities among participants who reported at least one gap (*N* = 124).

Overall, more participants reported one or more dissatisfaction-related gaps (*n* = 110, 48%) than non-engagement despite interest gaps (*n* = 65, 31%). Both gap types were significantly associated with loneliness; gaps reflecting not engaging in an activity despite being interested in doing so were moderately correlated with loneliness (*r* = −0.273, *p* < 0.001), and gaps reflecting engagement in an activity accompanied by dissatisfaction showed a similar association (*r* = −0.285, *p* < 0.001). The correlation between the two gap types was relatively weak (*r* = 0.186, *p* = 0.007), supporting their conceptual distinction, indicating that both are associated with loneliness.

**Figure 4:**
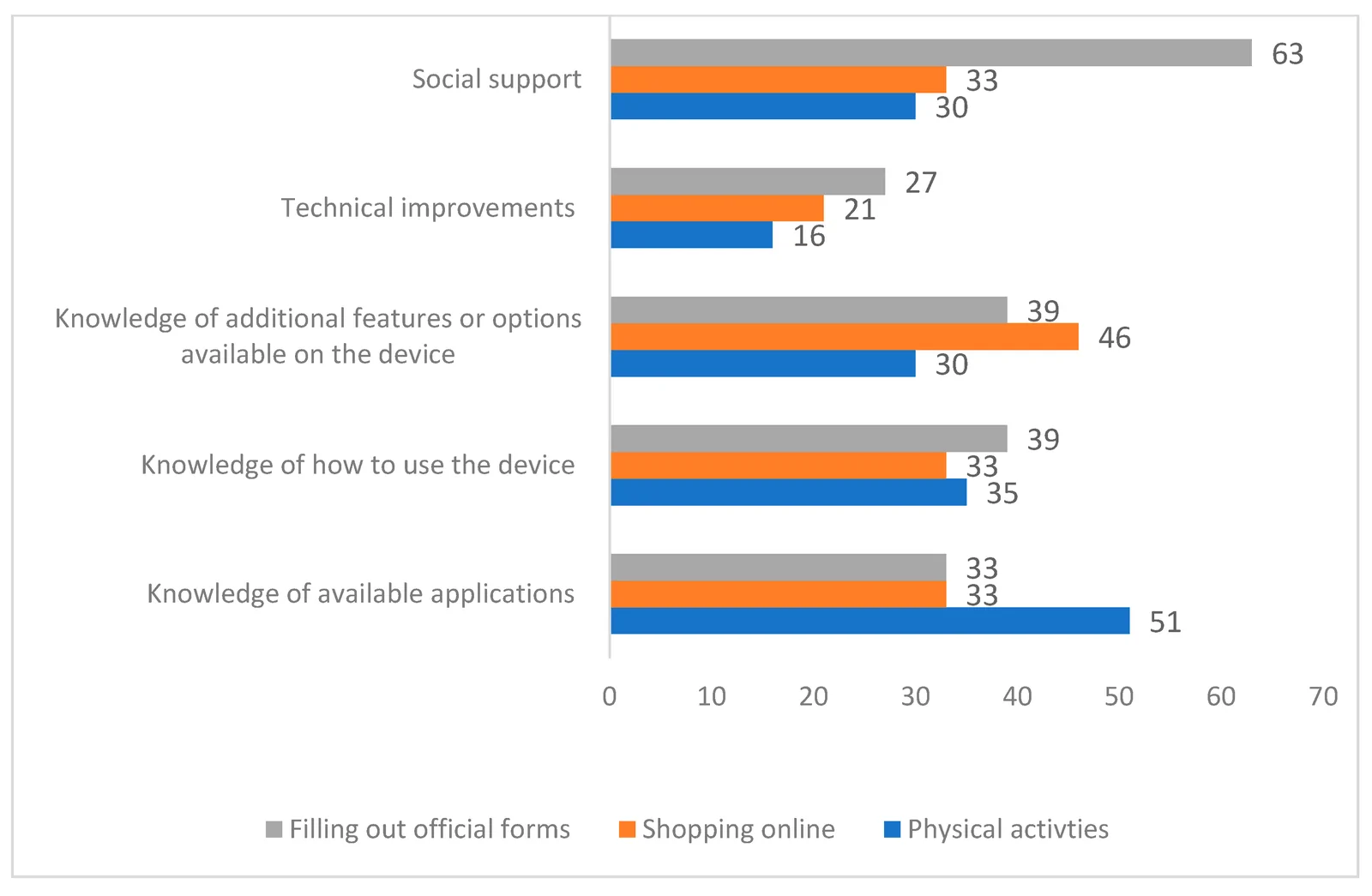
Percentages for the types of support required to overcome technology gaps related to three activities: filling out official forms, shopping online, and physical activity.

Figure 4 illustrates the types of support participants identified as necessary to bridge their technological gaps.

For physical activities, the most frequently mentioned needs were knowledge about available applications, social support for filling out official forms, and understanding the additional features or options offered for shopping online.

### 3.4. Correlations Between Loneliness, Depressive Symptoms, Sleep Quality, Functional Cognition, Sensorimotor Abilities, and Perceived Technology-Use Gaps

Table 4 presents the significant correlations among loneliness, depressive symptoms, sleep quality, cognitive function, sensorimotor abilities, and technology use.

As shown in Table 4, perceived technology-use gaps demonstrate small-to-moderate correlations with all study variables (−0.32 < *r* < 0.39). In addition, moderate correlations were observed between loneliness and depressive symptoms (*r* = −0.64) and between loneliness and the DLQ Parts A (*r* = −0.48) and B (*r* = −0.47). A particularly strong correlation was found between the DLQ Parts A and B (*r* = 0.82), indicating substantial conceptual overlap between the two measures. Therefore, only the domain that most directly represented functional cognition (Part B) was retained in the final model. Similar use of the DLQ Part B as an indicator of functional cognition has been reported in previous SEM research (Rosenblum & Cohen Elimelech, 2024).

### 3.5. Factors Associated with Perceived Loneliness: An SEM Analysis

**Figure 5:**
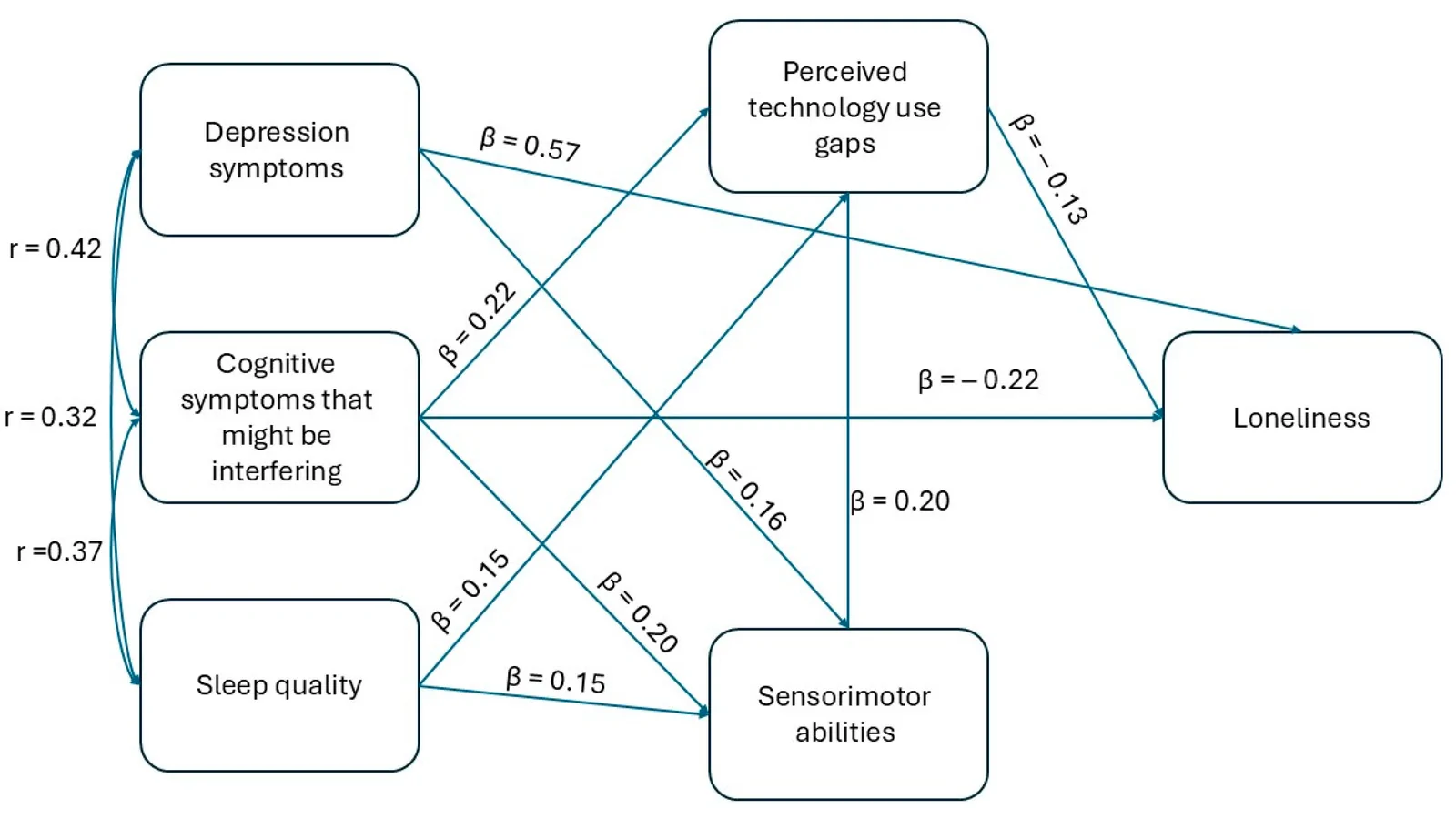
Structural Equation Model: factors associated with loneliness in older adults (standardized path coefficients, β). Note. Single-headed arrows represent path coefficients (β). Double-headed curved arrows represent correlations (r) between variables. All paths shown are statistically significant (p<0.05).

The SEM demonstrated an adequate fit to the data, χ^2^ (6) = 11.80, *p* = 0.067, comparative fit index = 0.986, normed fit index = 0.973, goodness of fit index = 0.985, root mean square error of approximation = 0.068, and standardized root mean square residual = 0.025. Figure 5 presents the structural relationships as standardized path coefficients (β) and correlations among the study variables.

According to Figure 5, higher levels of depression were associated with greater loneliness (β = −0.57, *p* < 0.001). Similarly, greater cognitive symptoms and impairments were associated with greater loneliness (β = −0.22, *p* = 0.001). Because higher scores on the De Jong-Gierveld Loneliness Scale indicate less loneliness, negative coefficients indicate that higher symptom burden was associated with greater loneliness.

In addition, higher levels of depressive symptoms were also associated with greater perceived sensorimotor difficulties (β = 0.16, *p* = 0.011). Poorer sleep quality was associated with both greater technology-use gaps (β = 0.15, *p* = 0.024) and greater perceived sensorimotor difficulties (β = 0.15, *p* = 0.020). Furthermore, greater cognitive difficulties (β = 0.22, *p* = 0.001) and greater sensorimotor impairment (β = 0.20, *p* = 0.004) were associated with larger perceived technology-use gaps.

Perceived gaps in technology use explained the association between sensorimotor abilities and loneliness. Finally, greater technology use-gaps were associated with greater loneliness (β = −0.13, *p* = 0.043).

As shown in Table 5, based on unstandardized bootstrap estimates of the indirect effects, the mediation analyses indicated significant indirect effects of depressive symptoms, sleep quality, cognitive symptoms and impairment, and sensorimotor abilities on loneliness.

These effects were primarily mediated by perceived gaps in technology use. Specifically, greater cognitive symptoms and impairments, poorer sleep quality, and higher levels of depressive symptoms were associated with larger technology-use gaps, which in turn were associated with greater loneliness. Sensorimotor abilities also served as mediators, but only within serial mediation pathways that included technology-use gaps as an additional mediator.

## 4. Discussion

This study examined the role of technology use in mediating the interplay between loneliness and health-related factors, including depressive symptoms, sleep quality, functional cognition, and sensorimotor abilities. Although the findings showed moderate-to-low correlations among these variables, they point to a nuanced, multifaceted functional picture of how technology use relates to older adults’ overall health. These findings align with previous research suggesting associations between health-related factors and technology use among older adults (Yazdani-Darki et al., 2020). Considering these health-related factors together offers a more comprehensive view of daily functioning because they collectively relate to older adults’ capacity to engage in everyday activities, maintain independence, and participate in social and technological contexts (Elliot et al., 2014; Luijkx et al., 2015; Rosenblum & Elimelech, 2021; Shin et al., 2017).

Among the health-related factors examined, depressive symptoms showed the strongest direct association with loneliness. The magnitude of this association was more than twice as large as that observed for functional cognitive impairments, indicating that depressive symptoms played a particularly prominent role within the model. This finding is consistent with established literature on the bidirectional relationship between depression and loneliness in later life (Nuyen et al., 2020; Ward et al., 2023). Furthermore, it highlights the centrality of affective states in shaping older adults’ sense of social connectedness.

Although the proposed model initially included sensorimotor abilities alongside other health-related factors, the findings suggest a more functionally specific role for this construct. As illustrated in the results, sensorimotor abilities were positioned adjacent to perceived gaps in technology use, suggesting a closer relevance to the usability of technological tools. This shift indicates that sensorimotor abilities are associated with loneliness primarily through their relationship with perceived technology-use gaps. Acknowledging that cognitive abilities are also related to the ability to use technology (Choi et al., 2021), maintaining a distinction between cognitive and sensorimotor abilities is consistent with existing literature (Campos et al., 2022) and may allow the model to reflect their potentially different roles more accurately.

Specifically, perceived gaps in technology use represent a potential mechanism linking health-related factors and loneliness. Although previous studies have primarily focused on the extent of technology use (Chopik, 2016; Fang et al., 2019; Zhang et al., 2024), the present findings highlight that the subjective experience of unmet technological needs may offer an additional perspective on loneliness. These perceived gaps comprise two distinct types: (a) a lack of engagement in desired technological activities despite an interest in doing so and (b) participation in such activities accompanied by dissatisfaction with their use. Both types underscore the importance of meaningful engagement in activities, which has implications for individuals’ ability and motivation to perform them (Myllykangas et al., 2002).

Therefore, technology-use gaps capture an aspect of everyday participation that is not reflected by measures of technology ownership or frequency of use alone. Such discrepancies are particularly relevant to loneliness because they represent barriers to participating in activities that individuals perceive as meaningful or socially valuable. This interpretation is further supported by a large-scale study, which found that although device ownership was associated with reduced loneliness, active and diverse engagement in digital activities demonstrated a significantly stronger association (Lee et al., 2025). Therefore, these findings suggest that merely owning a device may be insufficient; rather, the extent to which individuals can actively and satisfactorily engage with technology in meaningful activities may be more consequential.

According to the findings, the activities for which older adults most frequently reported perceived gaps were physical (30%), completing official forms (27%), and conducting online shopping (19%). Further, the findings indicate that different types of information are required to address technology-use gaps depending on the activity. Specifically, to support engagement in physical activities, most participants reported a need for greater awareness of available applications. In contrast, for online shopping and completing official forms, the most commonly cited needs related to understanding additional features or available options.

These findings are consistent with prior research demonstrating that older adults exhibit highly diverse and dynamic preferences in technology use to support daily activities (Booth et al., 2006; Zhao et al., 2024). Although the findings of the present study highlight potential domains for intervention, they also underscore substantial individual variability. Such variability emphasizes the importance of moving beyond generic approaches to examining and addressing each individual’s unique technology use preferences and perceived gaps (Vargo et al., 2021). Rather than simply assessing whether individuals use technology, it may be more informative to explore these perceived gaps—an approach uniquely captured by the DTUG questionnaire developed for the present study.

## 5. Limitations

Although this study offers relevant insights into older adults’ health-related factors, loneliness, and technology use, several limitations should be acknowledged. First, participant recruitment was conducted through an online platform, which required a basic level of technological ability. As a result, the study sample likely represents a more technologically engaged population, a conclusion further supported by the high rates of smartphone ownership and daily technology use observed in the study. Older adults with the greatest technology-related difficulties or the largest technology-use gaps may therefore have been underrepresented. Although participants reported a moderate need for adaptation, the high standard deviation suggests substantial heterogeneity in user needs, indicating that some individuals in the sample may still require adaptation to optimize technology use. Consequently, the findings should be interpreted as reflecting older adults who are already engaged with digital technologies and may not fully generalize to individuals with more limited access to, experience with, or confidence in technology.

Second, the study relied on self-reported questionnaires, which may limit the accuracy of the findings and may not fully capture actual functional abilities in real-world contexts. This limitation is particularly relevant for the assessment of functional cognition and sensorimotor abilities. The DTUG is a newly developed measure, and its internal consistency estimates should be considered preliminary. Additional studies are needed to further evaluate its reliability and validity and to examine its dimensional structure across diverse samples. Finally, this study’s cross-sectional design limits the ability to draw conclusions about causal relationships among technology use, loneliness, and health-related factors. Future research would benefit from including participants with a wider range of technological abilities, incorporating objective assessments of functioning, and employing longitudinal designs to better examine causal pathways over time.

## 6. Conclusions

This study brings attention to the experience of loneliness among older adults, an issue of growing global concern for this population. It highlights the potential role of technology use in supporting older adults in addressing loneliness. To our knowledge, this is the first study to conceptualize technology-use gaps as a potential mediating construct within the associations between functional and health-related factors and loneliness. As technology continues to evolve, focusing on daily activities provides a perspective that remains relevant over time. By emphasizing unmet needs in the use of technology for everyday activities and examining associations between health-related factors and loneliness, the findings point to opportunities to develop more targeted and responsive interventions. Additionally, these findings underscore the importance of addressing not only access to technology, but also the ability to engage with it in a meaningful and satisfying manner. Accordingly, interventions may benefit from identifying specific gaps in individuals’ technology use and targeting the underlying barriers rather than focusing on general technology instruction. These findings reflect older adults who were already able and willing to engage with digital technologies and should therefore be generalized with caution to populations with more pronounced technology-related barriers.

At a theoretical level, as technology becomes an increasingly integral part of everyday life, the findings highlight its importance in the experience of loneliness. Incorporating technology-related aspects into conceptualizations of loneliness, therefore, offers an additional lens for understanding loneliness as a human experience, particularly in later life.

## Figures and Tables

**Table 1:** Sociodemographic characteristics.

Characteristic	*M* (*SD*), Range
Age	70.55 (4.71), 65–87
Education years	15.10 (3.05), 8–30
	*n* (%)
Gender	
Female	104 (49)
Male	107 (51)
Marital status	
Currently in a relationship	155 (74)
Divorced	33 (16)
Widowed	11 (6)
Single	8 (4)
Employment status	
Retired	139 (66)
Employed	70 (33)
Unemployed	2 (1)
Time since retirement (yr) (*n* = 98)	
0–5	40 (41)
5–10	24 (24)
>10	34 (35)

**Table 2:** Loneliness, depressive symptoms, functional cognition, sensorimotor abilities and sleep quality.

Measure	*M* (*SD*), Range
Pittsburgh Sleep Quality Index	4.77 (2.81), 0–21
Daily Living Questionnaire	
Part A: Activities and participation	1.19 (0.30), 1–3.07
Part B: Cognitive symptoms that might be interfering	1.27 (0.37), 1–3.04
Washington Group Short Set on Functioning-Enhanced	1.27 (0.38), 1–4
De Jong-Gierveld Loneliness Scale	
Total score	41.77 (8.21), 17–55
Emotional loneliness score	22.26 (4.82), 6–30
Social loneliness score	19.50 (4.33), 5–25
	*n* (%)
Loneliness level (score range)	
Extremely lonely (11–22)	3 (1%)
Lonely (23–33)	35 (17%)
Moderately lonely (34–43)	83 (39%)
Not lonely (44–55)	90 (43%)
Geriatric Depression Scale	
Not typically cause for concern (0–4)	169 (80%)
Suggests mild depression (5–8)	22 (10%)
Suggests moderate depression (9–11)	14 (7%)
Suggests major depression (12–15)	6 (3%)

**Table 3:** Technology use patterns, abilities, and social support.

Technology Pattern ^1^	*n* (%)
Device ownership	
Owns a smartphone	206 (98%)
Owns a computer	176 (83%)
Owns a tablet	84 (40%)
Primary devices used ^2^	
Smartphone	127 (60%)
Computer	42 (20%)
Tablet	10 (5%)
	*M* (*SD*), Range
Utilization of devices over the past month (1 = not at all, 5 = several times per day)	4.95 (0.23), 2–5
Self-reported proficiency	
Knowledge: level of proficiency in using devices (1 = not good, 5 = excellent)	3.89 (0.99), 2–5
Adaptation: interested in receiving appropriate guidance to enhance device possibilities (1 = not at all, 5 = to a great extent)	2.44 (1.31), 1–5
Social support	
Opportunity to receive support from others (1 = not at all, 5 = to a great extent)	3.49 (1.26), 1–5
The level of support meets needs (1 = not at all, 5 = to a great extent)	3.61 (1.14), 1–5
Feeling comfortable asking for support (1 = not at all, 5 = to a great extent)	4.30 (0.93), 1–5
	*n* (%)
The person who supports	
Spouse	32 (15%)
Son or daughter	111 (53%)
Grandson or granddaughter	37 (17%)
Friend	35 (17%)

Note: ^1^ Participants could select more than one device; ^2^ Thirty-two (15.2%) did not respond to this question.

**Table 4:** Correlations among loneliness, depressive symptoms, sleep quality, functional cognition, sensorimotor abilities, and perceived technology-use gaps.

Variable	Loneliness	Depression	Sleep Quality	Functional Cognition—DLQ A: Activities and Participation	Functional Cognition—DLQ B: Cognitive Symptoms That Might Be Interfering	Sensorimotor Abilities
Depressive symptoms	−0.64 ***					
Sleep quality	−0.32 ***	0.29 ***				
Functional cognition—DLQ A: Activities and participation	−0.48 **	0.46 **	0.38 ***			
Functional cognition—DLQ B: Cognitive symptoms that might be interfering	−0.47 **	0.42 ***	0.37 ***	0.82 ***		
Sensorimotor abilities	−0.33 ***	0.36 ***	0.28 ***	0.53 ***	0.46 ***	
Perceived technology-use gaps	−0.32 ***	0.29 ***	0.31 ***	0.39 ***	0.36 ***	0.31 ***

** *p* < 0.01; *** *p* < 0.001. Note: Lower scores on the Loneliness Scale indicate higher levels of loneliness, whereas higher scores on all other measures indicate greater impairment or symptom severity.

**Table 5:** Mediation analysis based on unstandardized bootstrap estimates of indirect effects.

Path	Estimate	Lower Bound	Upper Bound	*p*
Depression → sensorimotor abilities → technology-use gap → loneliness	−0.004	−0.016	0.000	0.028
Cognitive symptoms and impairment → technology-use gap → loneliness	−0.231	−0.605	−0.038	0.017
Cognitive symptoms and impairment → sensorimotor abilities → technology-use gap → loneliness	−0.081	−0.250	−0.011	0.020
Sleep quality → technology-use gap → loneliness	−0.021	−0.063	−0.002	0.027
Sleep quality → sensorimotor abilities → technology-use gap → loneliness	−0.004	−0.018	0.000	0.038

## Data Availability

The data presented in this study are available on reasonable request from the corresponding author. The data are not publicly available due to ethical restrictions.

## Abbreviations

The following abbreviations are used in this manuscript:

ADL	Activities of Daily Living
DLQ	Daily Living Questionnaire
DTUG	Daily Technology-Use Gap
GDS	Geriatric Depression Scale
ICT	Information and Communication Technology
PSQI	Pittsburgh Sleep Quality Index
SEM	Structural Equation Modeling
WG-SS	Washington Group Short Set on Functioning
